# Evaluation of Podoplanin in Oral Leukoplakia and Oral Squamous Cell Carcinoma

**DOI:** 10.1155/2015/135298

**Published:** 2015-10-08

**Authors:** Ashok Patil, Kishor Patil, Suyog Tupsakhare, Mahesh Gabhane, Shrikant Sonune, Shilpa Kandalgaonkar

**Affiliations:** Department of Oral Pathology and Microbiology, SMBT Dental College and Hospital, Sangamner, Maharashtra 422608, India

## Abstract

*Background*. Recent studies have demonstrated that podoplanin was expressed in some dysplastic lesions adjacent to primary oral cancers suggesting that podoplanin expression may occur in early oral tumorigenesis and lymphangiogenesis and therefore is related to tumor growth. The purpose of this study is to determine the role of podoplanin as a biomarker for cancer risk assessment in oral leukoplakia and correlation of podoplanin expression with grades of oral squamous cell carcinoma (OSCC). *Materials and Methods*. In the present retrospective study, podoplanin expression was investigated immunohistochemically in 40 patients each of oral leukoplakia and OSCC. The scores were analyzed statistically using one-way ANOVA test followed by Tukey HSD. *Results*. By applying one-way ANOVA test, there was a highly significant increase of the podoplanin expression from mild to severe dysplasia and from well to poorly differentiated OSCC (*P* < 0.01). Statistically highly significant difference was present between scores of mild to moderate dysplasia, moderate to severe dysplasia, well to poorly differentiated OSCC, and moderately to poorly differentiated OSCC (Tukey HSD test, *P* < 0.01). *Conclusion*. Podoplanin can be used as a biomarker for early oral tumorigenesis and for malignant transformation risk assessment of premalignant lesions and as a tumor progression biomarker for advanced grades of OSCC.

## 1. Introduction

Oral cancer is a leading cause of cancer death and oral squamous cell carcinoma is the most common type of oral cancer. Early detection of high-risk premalignancy can decrease the morbidity and mortality associated with oral cancer [1]. Oral leukoplakia is the most prevalent premalignant lesion which shows histological diversity and has overall increased risk for development of invasive oral squamous cell carcinoma [2–4].

Multistep process with accumulation of genetic, epigenetic, and metabolic alterations resulting from exposure to carcinogens results in the development of cancer. The initiated and promoted cancer cells generally have a monoclonal feature. In the progression stage, it has heterogeneity due to the further genetic modifications of the cancer cells. Thus, biologically diverse patterns and clinically various behaviors are present with each OSCC. A number of these alterations have been identified recently which are helpful in understanding underlying biology of the disease [2, 5].

The mucin-type transmembrane glycoprotein podoplanin is a lymphatic-specific gene and has previously shown that podoplanin is a target gene of the homeobox gene Prox1, a master gene that controls the development of lymphatic progenitors from embryonic veins. Wetterwald in 1991 first reported the in vivo expression of podoplanin in lymphatic endothelium and named it as “E11 antigen.” It was further renamed as “podoplanin,” because of its low-level expression in kidney podocytes [6–9].

Lesions with dysplastic features are thought to be at higher risk of oral cancer but it was shown that some of the oral cancers developed from lesions that lacked dysplastic changes and also quality of the tissue, site of biopsy, and subjective factors like interobserver and intraobserver variation are a problem in the histologic assessment of epithelial dysplasia, presence, and severity. Therefore, additional objective markers are needed to identify high-risk lesions for proper management recommendations [2, 10–12].

Recent studies have demonstrated that podoplanin was expressed in some hyperplastic and dysplastic lesions adjacent to the primary oral cancers, suggesting that expression of podoplanin may occur in early oral tumorigenesis and may play a role in the malignant transformation [2, 10].

Malignant tumor cells in the oral area tend to metastasize in the relatively early stage to regional lymph nodes due to anatomical features of the oral cavity. Despite advances in diagnostic and therapeutic modalities, the prognosis of OSCC still remains poor; thus, it is essential to identify novel biomarkers of tumor progression that will facilitate treatment selection. The relationship of podoplanin in tumor invasion and its expression in human cancers indicate that it can be used as a biomarker for malignant transformation risk assessment and tumor progression biomarker for oral cancer [13–17].

The purpose of this study is to evaluate the expression of podoplanin in oral leukoplakia as a biomarker for cancer risk assessment and correlation of podoplanin with grades of OSCC.

## 2. Materials and Methods

### 2.1. Patients and Specimen

The study was carried out retrospectively on 40 previously diagnosed formalin fixed paraffin embedded tissue specimens, each of oral leukoplakia and OSCC. In 40 cases of oral leukoplakia, 20 were of mild dysplasia, 14 were of moderate dysplasia, and 6 were of severe dysplasia. In 40 cases of OSCC, 16, 16, and 08 were well-differentiated, moderately differentiated, and poorly differentiated, respectively. All 80 tissue specimens were stained along with appendix tissue sections as a positive control.

### 2.2. Immunohistochemistry

Primary antibody mouse IgG anti-podoplanin monoclonal antibody (clone D2-40) was used and staining was done by using the Dako Cytomation Envision plus peroxidase mouse system and diamino benzidine chromogen as substrate (Dako Cytomation).

Formalin fixed, paraffin embedded tissues were cut into 4 *μ*m sections and deparaffinized by using fresh xylene, followed by dehydration in graded alcohol. The sections were treated with peroxide block for 15 min and then antigen retrieval was done by using pressure cooker for 15 min. The tissue sections were then incubated with power block for 15 min. Tissue sections were further incubated in ready-to-use primary anti-podoplanin monoclonal antibody (clone D2-40), for 40 min. The sections were then incubated in super enhancer and with HRP at room temperature for 30 min each. After that, all the sections were incubated with diamino benzidine chromogen for 5 to 10 min. Lastly, the slides were washed and counterstained with hematoxylin. The negative control slides were stained similarly, except for the primary antibody.

### 2.3. Staining Interpretation

The cellular staining pattern for anti-D2-40 is cytoplasmic and sometimes membranous.

In oral leukoplakia, immunostaining was scored using a scoring system described by Kawaguchi et al. as follows [2]:

 0—if no expression was observed in any part of the epithelium.
 1—if expression was restricted to the basal layer of the epithelium (Figure 3(a)).
 2—if expression was observed in the basal and suprabasal layers at one area (Figure 3(b)).
 3—if the suprabasal layer expression was observed at two or three areas (Figure 3(c)).
 4—if the suprabasal layer expression was observed at more than three areas.

Score was calculated in 10 high power fields in each slide and mean was calculated per slide.

Calculation of cancer risk (according to De Vicente et al. [11] and Kawaguchi et al. [2]):

 Score 0-1: low risk or negative expression.
 Score 2 or more: high risk or positive expression.

In OSCC, podoplanin expression was scored as described by Rodrigo et al. by quantity of positive tumor cells on a scale of 0 to 5 as follows [17]:

 0: negative.
 1: less than 10% (Figure 4(a)).
 2: more than 10% and less than 30% (Figure 4(b)).
 3: more than 30% and less than 50%.
 4: more than 50% and less than 80% (Figure 4(c)).
 5: more than 80% positive staining.

Based on the staining intensity, positive specimens were classified into 4 categories [17]:

 3 = strong—dark brown staining of cells (Figure 4(c)).
 2 = moderate—staining between 2 extremes (dark brown and weak staining) (Figure 4(b)).
 1 = weak—faint staining (Figure 4(a)).
 0 = negative—no staining.

German Immunoreactive Score (IRS) was calculated by multiplying quantity score and staining intensity scores. Scores could range from 0 to 15:

 7 or higher = high reactivity and 0 to 6 = weak reactivity [17].

Score was calculated in 10 high power fields in each slide and mean was calculated per slide.

### 2.4. Statistical Analysis

The association between podoplanin expression status from mild to severe dysplasia in oral leukoplakia patients was analyzed using one-way ANOVA test, with the help of IBM SPSS statistics 20. Similarly, Podoplanin expression status from well to poorly differentiated OSCC was analyzed using one-way ANOVA test. The difference between the various groups was analyzed with the help of Tukey HSD test for both oral leukoplakia and OSCC.

## 3. Results

**Figure 1:**
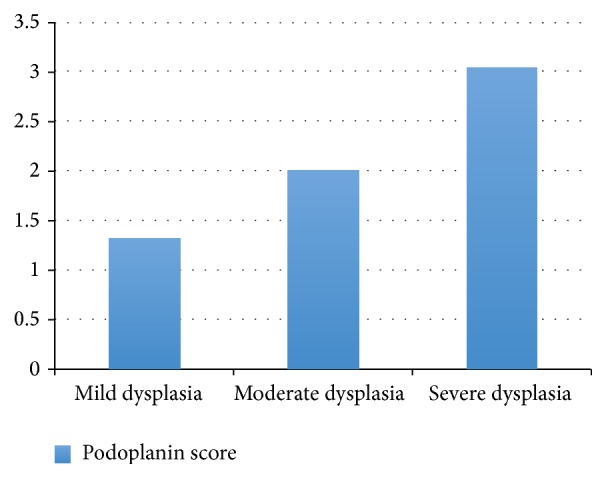
Graph showing increasing expression of podoplanin in oral leukoplakia as the grade of dysplasia increases.

**Figure 2:**
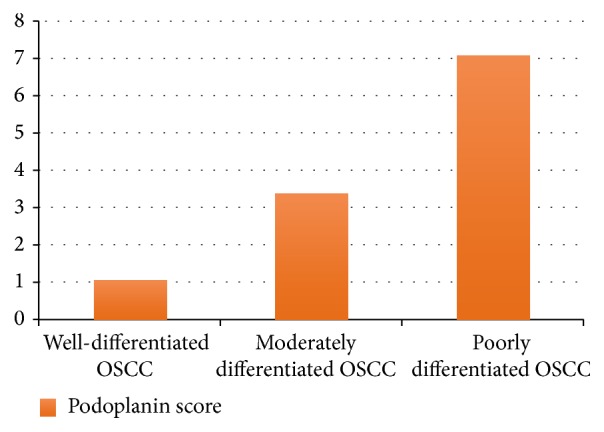
Graph showing increasing expression of podoplanin in OSCC as the grade of carcinoma increases.

**Figure 3:**
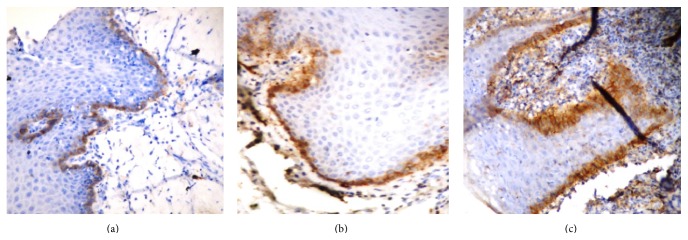
(a) Expression of podoplanin in oral leukoplakia: Score 1—expression restricted to basal layer of epithelium. (b) Expression of podoplanin in oral leukoplakia: Score 2—expression observed in basal and suprabasal layers at one area. (c) Expression of podoplanin in oral leukoplakia: Score 3—expression observed at two suprabasal areas.

**Figure 4:**
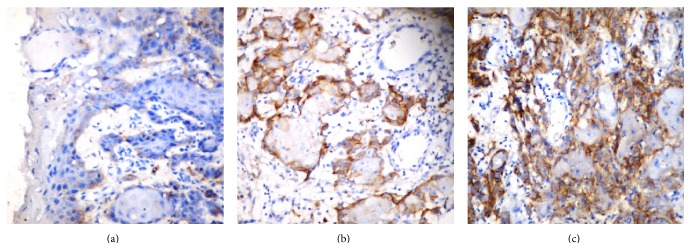
(a) Expression of podoplanin in OSCC: weak reactivity (>10% tumor cells positive and weak staining). (b) Expression of podoplanin in OSCC: moderate reactivity (10–30% tumor cells positive and moderate staining). (c) Expression of podoplanin in OSCC: high reactivity (50–80% tumor cells positive and strong staining).

By applying one-way ANOVA test, there was a highly significant increase of the podoplanin expression scores from mild (1.32 ± 0.43) to severe dysplasia (3.03 ± 0.33) (Table 1, Figure 1), as well as from well (1.03 ± 0.74) to poorly differentiated (7.07 ± 0.86) OSCC (*P* < 0.01) (Table 3, Figure 2). Also the increase of the podoplanin expression scores in between groups was analyzed using Tukey HSD test and a highly significant difference was shown between scores of the mild to moderate dysplasia, moderate to severe dysplasia, well to moderately differentiated OSCC, and moderately to poorly differentiated OSCC (*P* < 0.01) (Tables 2 and 4).

Thus, results show that there is increased podoplanin expression with increasing dysplasia in oral leukoplakia patients and also podoplanin expression score increases from well-differentiated OSCC to poorly differentiated OSCC.

Podoplanin expression and oral cancer risk for oral leukoplakia patients are as follows:

 Low risk/negative expression: Score 0 -1 = 16 patients (40%).
 High risk/positive expression: Score 2 or more = 24 patients (60%).

Immunoreactivity scoring for OSCC patients is as follows:

 0–6 = weak reactivity = 32 patients (80%).
 7 and more = high reactivity = 8 patients (20%).

## 4. Discussion

Although several markers have been proposed for diagnosing and predicting the behavior of dysplastic lesions, only few can be used as a biomarker for cancer risk assessment. Thus, identifying a new biomarker is of great clinical interest [14].

The upward clonal expansion of abnormal cells in the epithelial layers of dysplastic lesions and significantly higher risk of cancer development in such lesions was found in previous studies. The ability to detect these cells expanding beyond basal layers may allow us to visualize potential clonal expansion during tumorigenesis. Previously, it was shown that only a small and a phenotypically distinct subset of clonogenic cells is responsible for generating tumors and this subset of cells is considered as tumor initiating cells (TICs) or caner stem cells. Podoplanin is abnormally expressed in the early oral tumorigenesis and identified as a new marker for tumor initiating cells in squamous cell carcinoma [2, 11, 18].

In OSCC, podoplanin expression is restricted to invasive front. Recent studies have demonstrated that podoplanin mediates a pathway leading to collective and directional cell migration; and forced expression of podoplanin led to a dramatic change of cellular morphology. Also, adhesion and spreading of cells on the extracellular matrix protein fibronectin are enhanced by podoplanin expression. Induction of podoplanin expression results in multiple adjustments of intracellular signaling pathways and results in the modulation of Rho family GTPase activities, the phosphorylation of ERM (Ezrin, Radixin, and Moesin) proteins, and rearrangement of the actin cytoskeleton and enhances cell migration and invasion. Thus, podoplanin correlates with higher incidence of lymph node metastasis in early squamous cell carcinoma of the oral cavity and oropharynx [2, 11, 16, 19]. Recently several research studies have demonstrated that podoplanin seems to be expressed by aggressive tumors, with higher invasive and metastatic potential [8, 15, 20–22].

Recent studies had demonstrated that the expression of podoplanin correlates with the dysplasia in a grade dependent manner and a higher rate of malignant transformation [2]. It was previously shown that premalignant lesions with podoplanin expression at suprabasal layer may truly represent tumor initiating cells and with a higher risk of progression to invasive cancer [17, 23]. In the present study, podoplanin expression increases with the severity of dysplasia as it was found in previous studies of Kawaguchi et al. and Rodrigo et al. [2, 17]. We also found some mild dysplastic lesions showing increased podoplanin expression beyond basal layer. Previously, some studies also have not found the relation between the severity of epithelial dysplasia and malignant transformation. As podoplanin correlates with increased risk of malignant transformation; these lesions can be considered at higher risk of malignant transformation [11].

According to study done by Kawaguchi et al., 49% of patients had positive podoplanin expression in patients with dysplasia [2]. In the present study, 60% of patients with dysplasia had positive podoplanin expression. Taken together, these data not only support the potential importance of podoplanin in early oral tumorigenesis but also suggest that podoplanin can be used as a biomarker for evaluating malignant transformation risk in patients with premalignant lesions [1, 2].

In the present study, podoplanin positive cells were specifically located in most cases at the basal region of squamous cell carcinoma nests, close to the surrounding stromal cells, at invasive front, and the expression of podoplanin increases from well to poorly differentiated OSCC. Thus, it indicates that podoplanin expression increases with the increasing grades of OSCC. Recently, investigators have demonstrated that the invasion of squamous cell carcinomas in the area of head and neck does not depend upon cellular dedifferentiation, but on the acquisition of specific tumor cells which express podoplanin at the invasive front of the tumor. All these suggest that podoplanin may play some role in the regulation of differentiation, growth, and tumor progression of OSCC [6, 11, 14, 17, 24].

In the present study, a high expression of podoplanin was significantly correlated with increased dysplasia and advanced-stage OSCC; further studies on the correlation between the expression of podoplanin and prognosis-related factors in a larger sample population are needed. If the present study results could be supported by further studies, analysis of the podoplanin expression in the biopsied tissues could be helpful to clinicians and patients in evaluating the cancer risk in oral leukoplakia and as a biomarker for advanced grades of OSCC. Recently, various anti-podoplanin based therapeutic strategies, like anti-podoplanin CasMab, have been tried based on properties of podoplanin and thus can be useful to improve the prognosis of patients [8, 25, 26].

## 5. Conclusion

The present study concludes that the podoplanin can be used as a biomarker for early oral tumorigenesis and for malignant transformation risk assessment of premalignant lesions and as a biomarker for advanced grades of OSCC.

## Figures and Tables

**Table 1:** Expression of podoplanin in oral leukoplakia (one-way ANOVA).

Grades of epithelial dysplasia	*N*	Mean ± SD	*F* value	*P* value
Mild dysplasia	20	1.32 ± 0.43	49.53	<0.01
Moderate dysplasia	14	2.02 ± 0.30		
Severe dysplasia	06	3.03 ± 0.33		

SD = standard deviation. *P* < 0.01 = highly significant.

**Table 2:** Difference between podoplanin expression between various groups of oral leukoplakia (Tukey HSD test).

(*I*) Group	(*J*) Group	Mean difference (*I* − *J*)	*P* value
Mild dysplasia	Moderate dysplasia	0.7086	<0.01
Mild dysplasia	Severe dysplasia	1.713	<0.01
Moderate dysplasia	Severe dysplasia	1.005	<0.01

*P* < 0.01 = highly significant.

**Table 3:** Expression of podoplanin in OSCC (one-way ANOVA).

Grades of the SCC	*N*	Mean ± SD	*F* value	*P* value
Well-differentiated	16	1.03 ± 0.74	118.69	<0.01
Moderately differentiated	16	3.34 ± 0.97		
Poorly differentiated	08	7.07 ± 0.86		

SD = standard deviation. *P* < 0.01 = highly significant.

**Table 4:** Evaluation of the podoplanin expression between groups of OSCC (Tukey HSD test).

(*I*) Group	(*J*) Group	Mean difference (*I* − *J*)	*P* value
Well-differentiated	Moderately differentiated	2.037	<0.01
Well-differentiated	Poorly differentiated	5.769	<0.01
Moderately differentiated	Poorly differentiated	3.731	<0.01

*P* < 0.01 = highly significant.

## References

[1] Feng J.-Q., Mi J.-G., Wu L. (2012). Expression of podoplanin and ABCG2 in oral erythroplakia correlate with oral cancer development. *Oral Oncology*.

[2] Kawaguchi H., El-Naggar A. K., Papadimitrakopoulou V. (2008). Podoplanin: a novel marker for oral cancer risk in patients with oral premalignancy. *Journal of Clinical Oncology*.

[3] Warnakulasuriya S., Johnson N. W., van der Waal I. (2007). Nomenclature and classification of potentially malignant disorders of the oral mucosa. *Journal of Oral Pathology and Medicine*.

[4] Lee J. J., Hong W. K., Hittelman W. N. (2000). Predicting cancer development in oral leukoplakia: ten years of translational research. *Clinical Cancer Research*.

[5] Lee S. W., Park Y. W. (2012). Expression of endoglin and podoplanin in early and advanced oral squamous cell carcinoma. *Journal of the Korean Association of Oral and Maxillofacial Surgeons*.

[6] Schacht V., Dadras S. S., Johnson L. A., Jackson D. G., Hong Y.-K., Detmar M. (2005). Up-regulation of the lymphatic marker podoplanin, a mucin-type transmembrane glycoprotein, in human squamous cell carcinomas and germ cell tumors. *The American Journal of Pathology*.

[7] Wicki A., Lehembre F., Wick N., Hantusch B., Kerjaschki D., Christofori G. (2006). Tumor invasion in the absence of epithelial-mesenchymal transition: podoplanin-mediated remodeling of the actin cytoskeleton. *Cancer Cell*.

[8] Raica M., Cimpean A. M., Ribatti D. (2008). The role of podoplanin in tumor progression and metastasis. *Anticancer Research*.

[9] Al-Rawi M. A. A., Mansel R. E., Jiang W. G. (2005). Molecular and cellular mechanisms of lymphangiogenesis. *European Journal of Surgical Oncology*.

[10] Shi P., Liu W., Zhou Z.-T., He Q.-B., Jiang W.-W. (2010). Podoplanin and ABCG2: malignant transformation risk markers for oral lichen planus. *Cancer Epidemiology Biomarkers and Prevention*.

[11] De Vicente J. C., Rodrigo J. P., Rodriguez-Santamarta T., Lequerica-Fernández P., Allonca E., García-Pedrero J. M. (2013). Podoplanin expression in oral leukoplakia: tumorigenic role. *Oral Oncology*.

[12] Silverman S., Gorsky M., Lozada F. (1984). Oral leukoplakia and malignant transformation. A follow-up study of 257 patients. *Cancer*.

[13] Ohno F., Nakanishi H., Abe A. (2007). Regional difference in intratumoral lymphangiogenesis of oral squamous cell carcinomas evaluated by immunohistochemistry using D2-40 and podoplanin antibody: an analysis in comparison with angiogenesis. *Journal of Oral Pathology and Medicine*.

[14] Mashhadiabbas F., Mahjour F., Mahjour S. B., Fereidooni F., Hosseini F. S. (2012). The immunohistochemical characterization of MMP-2, MMP-10, TIMP-1, TIMP-2, and podoplanin in oral squamous cell carcinoma. *Oral Surgery, Oral Medicine, Oral Pathology and Oral Radiology*.

[15] Ohta M., Abe A., Ohno F. (2013). Positive and negative regulation of podoplanin expression by TGF-*β* and histone deacetylase inhibitors in oral and pharyngeal squamous cell carcinoma cell lines. *Oral Oncology*.

[16] Huber G. F., Fritzsche F. R., Züllig L. (2011). Podoplanin expression correlates with sentinel lymph node metastasis in early squamous cell carcinomas of the oral cavity and oropharynx. *International Journal of Cancer*.

[17] Rodrigo J. P., García-Carracedo D., González M. V., Mancebo G., Fresno M. F., García-Pedrero J. (2010). Podoplanin expression in the development and progression of laryngeal squamous cell carcinomas. *Molecular Cancer*.

[18] Atsumi N., Ishii G., Kojima M., Sanada M., Fujii S., Ochiai A. (2008). Podoplanin, a novel marker of tumor-initiating cells in human squamous cell carcinoma A431. *Biochemical and Biophysical Research Communications*.

[19] Miyazaki Y., Okamoto E., González-Alva P. (2009). The significance of podoplanin expression in human inflamed gingiva. *Journal of Oral Science*.

[20] Zygogianni A. G., Kyrgias G., Karakitsos P. (2011). Oral squamous cell cancer: early detection and the role of alcohol and smoking. *Head and Neck Oncology*.

[21] Martín-Villar E., Fernández-Munoz B., Parsons M. (2010). Podoplanin associates with CD44 to promote directional cell migration. *Molecular Biology of the Cell*.

[22] Toll A., Gimeno-Beltrán J., Ferrandiz-Pulido C. (2012). D2-40 immunohistochemical overexpression in cutaneous squamous cell carcinomas: a marker of metastatic risk. *Journal of the American Academy of Dermatology*.

[23] Inoue H., Miyazaki Y., Kikuchi K. (2012). Podoplanin promotes cell migration via the EGF-Src-Cas pathway in oral squamous cell carcinoma cell lines. *Journal of oral science*.

[24] Wicki A., Christofori G. (2007). The potential role of podoplanin in tumour invasion. *British Journal of Cancer*.

[25] Cîmpean A. M., Raica M., Izvernariu D. A., Tǎtucu D. (2007). Lymphatic vessels identified with podoplanin: comparison of immunostaining with three different detection systems. *Romanian Journal of Morphology and Embryology*.

[26] Kato Y., Kaneko M. K. (2014). A cancer-specific monoclonal antibody recognizes the aberrantly glycosylated podoplanin. *Scientific Reports*.

